# Delineation of the movement disorders associated with *FOXG1* mutations

**DOI:** 10.1212/WNL.0000000000002585

**Published:** 2016-05-10

**Authors:** Apostolos Papandreou, Ruth B. Schneider, Erika F. Augustine, Joanne Ng, Kshitij Mankad, Esther Meyer, Amy McTague, Adeline Ngoh, Cheryl Hemingway, Robert Robinson, Sophia M. Varadkar, Maria Kinali, Vincenzo Salpietro, Margaret C. O'Driscoll, S. Nigel Basheer, Richard I. Webster, Shekeeb S. Mohammad, Shpresa Pula, Marian McGowan, Natalie Trump, Lucy Jenkins, Frances Elmslie, Richard H. Scott, Jane A. Hurst, Belen Perez-Duenas, Alexander R. Paciorkowski, Manju A. Kurian

**Affiliations:** From Molecular Neurosciences (A.P., J.N., E.M., A.M., A.N., S.S.M., B.P.-D., M.A.K.), Developmental Neurosciences Programme, University College London–Institute of Child Health; Departments of Neurology (A.P., C.H., R.R., S.M.V., M.A.K.) and Neuroradiology (K.M.), Department of Molecular Genetics, North East Thames Regional Genetics Services (N.T., L.J.), and Department of Clinical Genetics (R.H.S., J.A.H.), Great Ormond Street Hospital for Children NHS Foundation Trust, London, UK; Department of Neurology (R.B.S., E.F.A., A.R.P.), Center for Human Experimental Therapeutics (E.F.A.), and Departments of Pediatrics and Biomedical Genetics (A.R.P.), University of Rochester Medical Center, NY; Gene Transfer Technology Group (J.N.), UCL–Institute for Women's Health, London; Departments of Paediatric Neurology (M.K., V.S.) and Paediatrics (M.C.O.), Chelsea and Westminster NHS Foundation Trust, London; Department of Perinatal Neurology (S.N.B.), Hammersmith Hospital, London, UK; Institute for Neuroscience and Muscle Research (R.I.W.), Department of Neurology (R.I.W.), and Neuroimmunology Group, Institute for Neuroscience and Muscle Research (S.S.M.), The Children's Hospital at Westmead, Sydney, Australia; Child Development Centre (S.P., M.M.) and South West Thames Regional Genetics Service (F.E.), St George's University Hospitals NHS Foundation Trust, London, UK; and Department of Child Neurology (B.P.-D.) and Centre for Biomedical Research in Rare Diseases (CIBERER-ISCIII) (B.P.-D.), Hospital Sant Joan de Déu, Universitat de Barcelona, Spain.

## Abstract

**Objective:**

The primary objective of this research was to characterize the movement disorders associated with *FOXG1* mutations.

**Methods:**

We identified patients with *FOXG1* mutations who were referred to either a tertiary movement disorder clinic or tertiary epilepsy service and retrospectively reviewed medical records, clinical investigations, neuroimaging, and available video footage. We administered a telephone-based questionnaire regarding the functional impact of the movement disorders and perceived efficacy of treatment to the caregivers of one cohort of participants.

**Results:**

We identified 28 patients with *FOXG1* mutations, of whom 6 had previously unreported mutations. A wide variety of movement disorders were identified, with dystonia, choreoathetosis, and orolingual/facial dyskinesias most commonly present. Ninety-three percent of patients had a mixed movement disorder phenotype. In contrast to the phenotype classically described with *FOXG1* mutations, 4 patients with missense mutations had a milder phenotype, with independent ambulation, spoken language, and normocephaly. Hyperkinetic involuntary movements were a major clinical feature in these patients. Of the symptomatic treatments targeted to control abnormal involuntary movements, most did not emerge as clearly beneficial, although 4 patients had a caregiver-reported response to levodopa.

**Conclusions:**

Abnormal involuntary movements are a major feature of *FOXG1* mutations. Our study delineates the spectrum of movement disorders and confirms an expanding clinical phenotype. Symptomatic treatment may be considered for severe or disabling cases, although further research regarding potential treatment strategies is necessary.

*FOXG1* (located on chromosome 14q12)^1^ has a crucial role in the development of the fetal telencephalon and is primarily involved in promoting neural precursor proliferation and cerebral cortical expansion.^2^ *FOXG1* continues to be expressed in neurons postnatally and through adulthood and has been linked with promoting survival of postmitotic neurons.^3^ Mutations in *FOXG1* produce a distinct phenotype^4–6^ typically manifesting in infancy and early childhood with acquired microcephaly, epilepsy, motor and cognitive delay, severe intellectual disability and absent language.^7^ Neurodevelopmental delay is a presenting feature,^1^ often accompanied by poor feeding, irritability, hypotonia, and visual inattention. Epilepsy of varying severity presents in infancy (often as early-onset epileptic encephalopathy) or childhood. Multiple seizure types (including generalized tonic-clonic, myoclonic, and complex partial seizures with or without generalization) have been associated with *FOXG1* mutations.^8^ Delayed myelination, frontotemporal abnormalities, and corpus callosum abnormalities are often identified on brain imaging.^6,8,9^

Abnormal involuntary movements have been reported in FOXG1 syndrome,^10^ but they have not been characterized in detail. The objective of this study was to define the *FOXG1*-associated movement disorder phenotype, examine functional impact, and describe the caregiver-reported value of available treatments.

## METHODS

### Standard protocol approvals, registrations, and patient consents.

This study was approved by the University of Rochester Research Subjects Review Board (RSRB43415), the National Research Ethics Service in the United Kingdom (National Research Ethics Service Committee: London–Bloomsbury, REC reference: 13/LO/0168, IRAS project ID: 95005), and Great Ormond Street Hospital Research and Development Audit Department (reference: 15NM32). Written informed consent was obtained from all guardians of participants. Consent to disclose was obtained from the guardians of all participants identifiable on video footage.

### Patient ascertainment.

Twenty-eight patients with mutations in *FOXG1* were identified as part of ongoing studies of developmental brain disorders. Sixteen were ascertained through the Genetic Studies of Developmental Brain Disorders research program (Rochester, NY) and 12 either through the Study of Inherited Metabolic Diseases program (London, UK) or through UK clinical services. Details of the epilepsy and neurodevelopmental outcome of 18 of 28 patients have been previously published.^7,8^

### Genetic analysis.

All patients were diagnosed with *FOXG1* mutations as part of their routine clinical care using clinically available testing, through (1) chromosomal microarray studies identifying gene deletions/duplications, (2) targeted *FOXG1* gene sequencing, or (3) a diagnostic multiple gene panel for early infantile epileptic encephalopathy and severe neurodevelopmental delay.^11^ Although *FOXG1 cis*-regulatory elements have been hypothesized to be present in the region distal to the gene,^7,12,13^ patients with 14q12 microdeletions that did not encompass *FOXG1* were not included in this study.

### Movement disorders.

Characterization of the movement disorders, through direct clinical examination and/or evaluation of video footage, was possible for 25 of 28 patients. In 13 of 25 cases, movements were assessed in person by investigators. Videos of sufficient quality were obtained from 17 patients. These were independently rated by 3 different teams of movement disorder specialists and consensus agreement was reached. Information from both sources was utilized for the characterization of movement disorders. Observed movements were classified according to established criteria.^14^ For all 25 patients, a detailed retrospective review of medical records and clinical investigations was performed. Sixteen caregivers completed a telephone-administered questionnaire regarding abnormal involuntary movements and perceived response to medication.

## RESULTS

### Genetics.

Most *FOXG1* mutations occurred de novo, with the exception of those found in siblings (table 1, table e-1 on the *Neurology*® Web site at Neurology.org), inherited from clinically unaffected parents with presumed somatic mosaicism. A wide variety of mutations were reported, 6 of which (c.572T>G, p.Met191Arg; c.695A>G, p.Asn232Ser; c.946del, p.Leu316Cysfs*10; c.981C>A, p.Tyr327*; c.1186C>A, p.Cys396*; c.263_278del16) have not been previously reported in the literature (table 1 and table e-1).

**Table 1:**
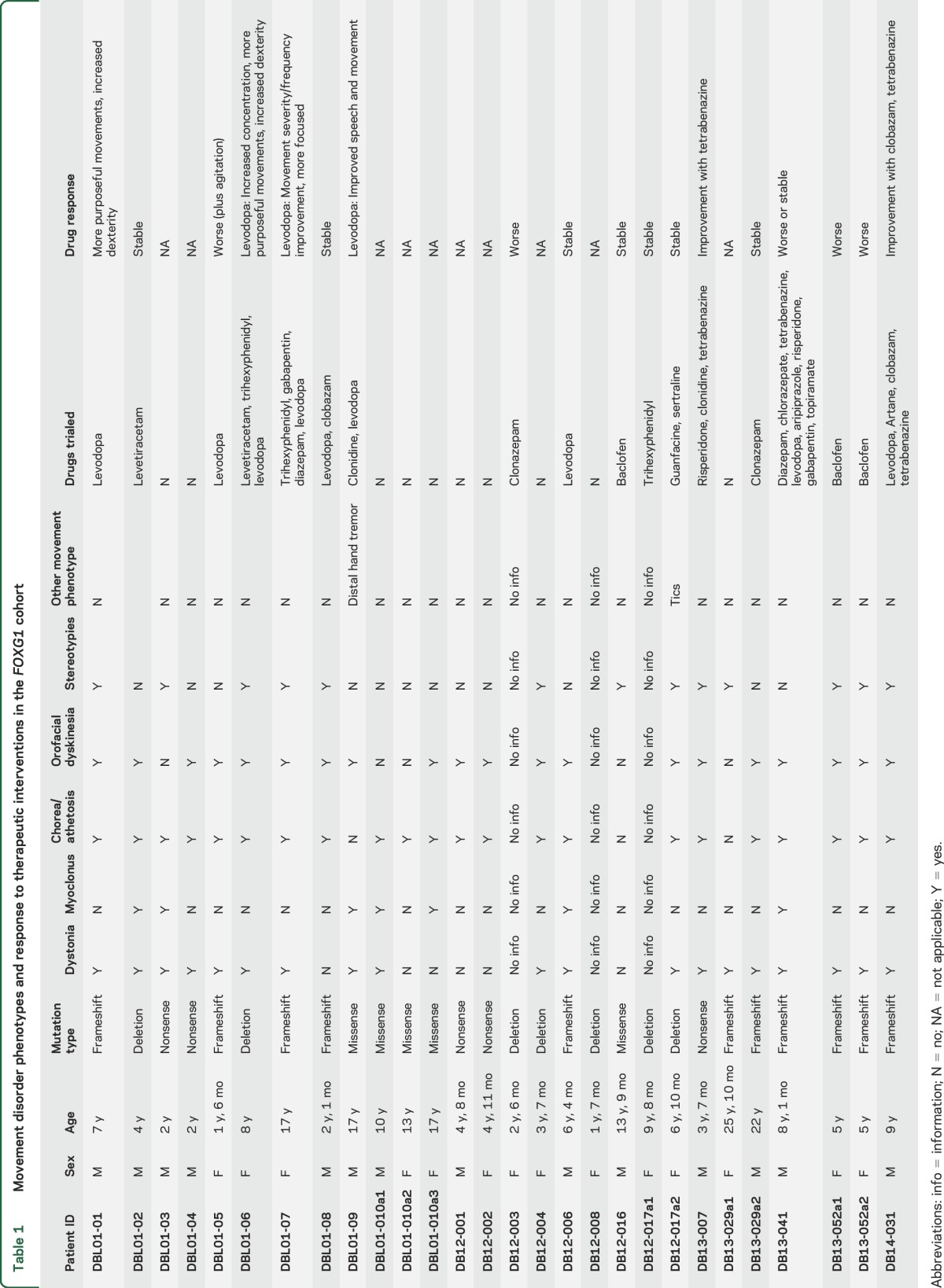
Movement disorder phenotypes and response to therapeutic interventions in the *FOXG1* cohort

### General clinical, radiologic, and biochemical features.

Within the cohort, patient age ranged from 18 months to 25 years (median 6 years, 7 months; mean 8 years, 4 months). The sexes were equally represented, with 14 male and 14 female patients. Microcephaly, defined as greater than 2 SDs below the mean for age, was present in 85% (23/27) and acquired postnatally in most cases (table e-2). Seventy-nine percent of patients (22/28) were diagnosed with epilepsy, and of the remaining 6 patients, 2 had a history of febrile convulsions (table e-2). Neurodevelopmental delay was present in all cases. Notably, 4 of 5 patients with missense mutations had a relatively mild phenotype, manifesting normal head growth, independent ambulation, spoken language, and purposeful hand function. The fifth patient (patient ID DB12-016) with a missense mutation (c.577G>A; p.Ala193Thr) had a phenotype more similar to classic FOXG1 disorder, although he did have a relatively mild movement disorder consisting only of stereotypies. In contrast to those with missense mutations, patients harboring large-scale deletions, frameshift variants or nonsense mutations tended to be more severely affected, with a phenotype more consistent with the classically described FOXG1 disorder (table e-2).^1,7^ MRI brain scans were available for review in 21 of 28 patients (figure e-1, table e-2). Common findings included corpus callosum abnormalities (86%, 18/21 patients), frontal or frontotemporal underdevelopment (71%, 15/21 patients) and mild cerebellar hypoplasia (43%, 9/21 patients). Myelin maturation was assessed in 12 patients, 9 of whom showed delayed myelination. No obvious radiologic basal ganglia abnormalities were seen. CSF neurotransmitter analysis was undertaken in 12 patients. Abnormalities, namely, low homovanillic acid (HVA) and 5-hydroxyindoleacetic acid (5-HIAA), were seen in 17% of patients (2/12) (table e-2).

### Movement disorder phenotypes.

Abnormal involuntary movements were present in all patients with available data on involuntary movements (n = 25) (table 1, videos 1–8). Chorea/athetosis (88%, 22/25 patients) (video 1), orolingual/facial dyskinesias (80%, 20/25), and dystonia (76%, 19/25) (video 2) were most frequently present. Orolingual/facial dyskinesias were often prominent and included forehead wrinkling, grimacing, lip pursing, jaw opening, and tongue protrusion (video 3). Facial dystonia was also featured in 2 cases (DB13-029a1 and DB13-029a2) (video 4). Stereotypies (video 5) were present in 52% of patients (13/25) and more commonly involved the upper limbs with mouthing of toys, grasping clothes or objects, nail biting and, rarely, midline wringing. Stereotypies in the lower limbs (pulling, pedaling), trunk (body rocking) and mouth (bruxism) were seen less frequently. Myoclonus was observed in 28% of patients (7/25). Myoclonus and dystonia were both present in 24% (6/25) (video 6). Tremor (video 7) and tics were each identified in one patient. Pyramidal features were also commonly reported, and many patients were noted to have axial/peripheral hypotonia, brisk deep tendon reflexes, upgoing plantar responses, and ankle clonus.

Sixteen families completed a parent-proxy questionnaire about involuntary movements (table e-3). The presence of abnormal involuntary movements was recognized by family members in 100% of patients (16/16), developing by 12 months of age in all but one case, in which the age at onset was unknown (table e-3). While the abnormal movements remained relatively stable in 50% of patients (8/16), for approximately half (44%, 7/16), the movement disorder became progressively worse over the disease course. Movements were described as generalized in 75% of patients (12/16) and were universally functionally impairing, interfering with toileting, dressing, sleeping, eating, playing, learning and nonverbal communication. Overall in our cohort, only 1 of 28 patients was hospitalized specifically for management of their movement disorder, although none presented with status dystonicus.

Medications prescribed to manage the movement disorder were often not effective per the report of caregivers. A number of medications (table 1) were trialed in 18 of 28 patients, with no obvious benefit in many cases. Worsening of abnormal involuntary movements or intolerable side effects were reported in 28% of cases (5/18). Clobazam was reported as helpful in 1 of 2 patients and tetrabenazine in 2 of 3 patients. Levodopa provided benefit in 4 of 9 patients. Two of the levodopa responders were noted to have an improvement in abnormal involuntary movements, whereas 2 had an improvement in overall dexterity and upper limb function but no obvious change in abnormal involuntary movements. Response to levodopa was particularly evident in patient DBL01-09, who had low CSF levels of HVA and 5-HIAA indicating impaired dopamine and serotonin turnover. This patient had a significant reduction in drooling, dysarthria, dystonia and hand tremor (video 7) and, also, improvement of speech and language function. The remainder of the levodopa responders (patients DBL01-01, DBL01-06, and DBL01-07) had normal CSF neurotransmitter levels. Patient DBL01-08, who had low CSF HVA and biopterin levels, did not report any obvious benefit after levodopa administration.

## DISCUSSION

Abnormal involuntary movements were present in 100% of our cohort of patients with *FOXG1* mutations, which supports findings from recent studies that movement disorders are a cardinal feature of this disorder.^13,15^ Our study clearly demonstrates that FOXG1 syndrome is associated with a wide spectrum of predominantly hyperkinetic movement disorders, most frequently generalized chorea, distal athetosis, dystonia and orolingual/facial dyskinesias.

Stereotypies were reported in more than half the cases. We observed a number of non-midline stereotypies mainly with hand separation, which were both symmetrical and asymmetrical in nature (video 5). Repetitive finger movements, pulling, grasping, touching, and stroking, as well as lower limb pedaling, were frequently seen.

Myoclonic jerks were also seen in some patients. In 2 of 5 patients with missense *FOXG1* mutations, the combination of myoclonus and dystonia was a prominent clinical feature (videos 6–8), and reminiscent of *SGCE* mutation–positive myoclonus-dystonia syndrome (MDS) caused by *DYT11* mutations.^16–18^ MDS is reported to show genetic heterogeneity,^17^ and we propose that *FOXG1* mutations should be included in the differential diagnosis when investigating *SGCE* mutation–negative MDS, especially in the context of neurodevelopmental delay.

Variability in movement disorder symptoms and severity was observed in our patients with missense mutations. Patient DBL12-016 exhibited only stereotypies (table 1). Four patients with missense mutations (DBL01-09, DBL01-10a1, DBL01-10a2, DBL01-10a3) had a strikingly milder general clinical presentation, less typical MRI findings (table e-2), and the hyperkinetic movement disorder was the most prominent and disabling clinical feature. Intrafamilial differences were also evident in siblings DBL01-10a1, DBL01-10a2, and DBL01-10a3 (video 8, table 1). The underlying basis for such variability is currently unclear but *FOXG1* genotype as well as additional genetic, epigenetic or environmental factors may be contributory.

The involuntary abnormal movements were universally described as functionally impairing in the subset of families who completed the telephone-based questionnaire. It is, therefore, not surprising that many patients tried a number of different medications to suppress the involuntary movements. Little has been reported about the efficacy of therapeutic agents for *FOXG1*-related movement disorders. Within our cohort, the majority of medications prescribed to manage abnormal movements were reported by caregivers to be nonbeneficial, worsen overall function or cause intolerable side effects. Two perhaps notable exceptions were tetrabenazine and levodopa, which led to clinical improvement in some patients. We found associations of *FOXG1* mutations with secondary CSF neurotransmitter abnormalities, which have been described in several neurometabolic disorders including *MECP2* mutations and early-onset epileptic encephalopathies.^19,20^ Our observations suggest that further studies are necessary to determine the role of monoamines and related drugs in this condition, in order to optimize treatment regimens for patients with *FOXG1* mutations. Furthermore, while more clinical data are needed to further evaluate drug efficacy in patients with *FOXG1* mutations, our data suggest that a trial of levodopa or tetrabenazine could be considered for the symptomatic treatment of patients with *FOXG1* mutations who have prominent or disabling dystonia or dyskinesia.

FOXG1 has an important role in fetal telencephalon development but its function in postmitotic neurons is less clear. Human adult brain transcriptional atlas data^21^ indicate that FOXG1 is highly expressed in the basal ganglia (especially in the putamen and caudate) compared to many other brain areas (http://human.brain-map.org).^22^ Many studies suggest that FOXG1 has a postulated role in regulating neuronal death.^1,23^ Recent studies in mice^24^ suggest that *FOXG1* mutations cause overexpression of a group of genes in the basal ganglia that are involved in movement control, although no models directly examining neuronal function or survival in the striatum currently exist. Neuronal dysfunction or early apoptosis within the basal ganglia could explain the movement disorders and aberrant neurotransmitter patterns seen in this condition. However, the mechanism by which mutations in *FOXG1* and other related genes cause disease is yet to be fully elucidated and likely to be multifactorial.

We were able to well characterize the phenotypic spectrum of movement disorders associated with *FOXG1* mutations. However, given the retrospective nature of our study, there are limitations to our approach. Participants were identified as having a *FOXG1* mutation as part of their clinical care, and not in the context of a genetic epidemiology study. As such, our cohort may show selection bias and not be representative of the full *FOXG1* mutation spectrum. Despite these limitations, we still portray a broad phenotypic spectrum including both mildly and severely affected patients. As a retrospective study, there was no standardized approach to videotaping. Video recordings were not standardized, and the majority of videos were filmed in the home setting by family members. As a result, for some cases, our clinical assessment was limited by video quality and the presence of different movement disorders (particularly episodic movements that may not have been captured in the videos) may have been underestimated. The use of caregiver-reported ratings for assessing response to treatment represents a potential source of bias and is less rigorous than clinical assessment of treatment response. Nevertheless, this is the most comprehensive analysis of the movement disorder associated with *FOXG1* mutations to date.

Our research confirms that movement disorders are a defining feature of patients with *FOXG1* mutations. Given the expanding disease spectrum, *FOXG1* genetic analysis should be considered in the differential diagnosis for patients with unexplained hyperkinesia, especially in the context of neurodevelopmental delay. While further research is required, our study suggests that tetrabenazine and levodopa may be effective in some cases. Further elucidation of disease mechanisms in FOXG1 syndrome is paramount in order to provide pathogenic insight into the processes governing movement disorders, epileptogenesis and neurodevelopmental delay, thereby potentially identifying novel therapeutic targets for future translational research.

## Supplementary Material

Data Supplement

Videos

## GLOSSARY

HVA: homovanillic acid
MDS: myoclonus-dystonia syndrome
