# Supplementary material for: Delineation of the movement disorders associated with FOXG1 mutations
Source: Neurology. 2016 May 10;86(19):1794–800. doi: 10.1212/WNL.0000000000002585 (PMC4862244; doi:10.1212/WNL.0000000000002585)
Supplement: Data Supplement [file supp_86_19_1794__index.html]

Data Supplement 

# Delineation of the movement disorders associated with *FOXG1* mutations

## Data Supplement

Three tables and one figure; two PDF files.

**Neurology® data supplements are not copyedited before publication. Published editorials and translations have been copyedited.  
 © 2016 American Academy of Neurology.  
  
 Files in this Data Supplement:**

- Tables e-1 to e-3 - PDF file
- Figure e-1 - PDF file
