# Supplementary material for: Delineation of the movement disorders associated with FOXG1 mutations
Source: Neurology. 2016 May 10;86(19):1794–800. doi: 10.1212/WNL.0000000000002585 (PMC4862244; doi:10.1212/WNL.0000000000002585)
Supplement: Data Supplement [file supp_WNL.0000000000002585_Tables.pdf]

Patient ID	*FOXG1* mutation	Effect on protein	Mutation type	De novo	Previously reported	Reference of previous mutation report
**DBL01-01**	c.735delC	p.Tyr246Thrfs*80	Frameshift	Y	Y	Seltzer et al 2014 [8]
**DBL01-02**	5.4kb deletion at Chr. 14q12 including *FOXG1*		Deletion	Y	N/A	-
**DBL01-03**	c.981C>A	p.Tyr327*	Nonsense	Y	N	-
**DBL01-04**	c.1186C>A	p.Cys396*	Nonsense	Y	N	-
**DBL01-05**	c.460dupG	p.Glu154Glyfs*301	Frameshift	Y	Y	Kortum et al. 2011 [7]
**DBL01-06**	2kb deletion at Chr. 14q12, including *FOXG1*		Deletion	Y	N/A	-
**DBL01-07**	c.460dupG	p.Glu154Glyfs*301	Frameshift	Y	Y	Kortum et al. 2011 [7]
**DBL01-08**	c.946del	p.Leu316Cysfs*10	Frameshift	Y	N	-
**DBL01-09**	c.695A>G	p.Asn232Ser	Missense	Y	N	-
**DBL01-010a1**	c.572T>G	p.Met191Arg	Missense	N (maternal somatic mosaicism)	N	-
**DBL01-010a2**	c.572T>G	p.Met191Arg	Missense	N (maternal somatic mosaicism)	N	-
**DBL01-010a3**	c.572T>G	p.Met191Arg	Missense	N (maternal somatic mosaicism)	N	-
**DB12-001**	c.651C>G	p.Y217*	Nonsense	Y	Y	Seltzer et al, 2014[8]
**DB12-002**	Clinical report did not include	p.Gln86*	Nonsense	Y	Y	Seltzer et al, 2014[8]
**DB12-003**	6 Mb del		Deletion	Y	Y	Seltzer et al, 2014[8]
**DB12-004**	1.1 Mb del		Deletion	Y	Y	Seltzer et al, 2014[8]
**DB12-006**	c.460dupG	p.Glu154Glyfs*301	Frameshift	Y	Y	Seltzer et al, 2014[8]
**DB12-008**	0.25 Mb del		Deletion	Y	Y	Seltzer et al, 2014[8]
**DB12-016**	c.577G>A	p.Ala193Thr	Missense	Y	Y	Seltzer et al, 2014[8]
**DB12-017a1**	c.515_577del63	p.Gly172_Met192del	Deletion	Likely parental gonadal mosaic	Y	Seltzer et al, 2014[8]
**DB12-017a2**	c.515_577del63	p.Gly172_Met192del	Deletion	Likely parental gonadal mosaic	Y	Seltzer et al, 2014[8]
**DB13-007**	c.586C>T	p.Gln196*	Nonsense	Y	Y	Seltzer et al, 2014[8]
**DB13-029a1**	c.460dupG	p.Glu154Glyfs*301	Frameshift	Likely parental gonadal mosaic	Y	Seltzer et al, 2014[8]
**DB13-029a2**	c.460dupG	p.Glu154Glyfs*301	Frameshift	Likely parental gonadal mosaic	Y	Seltzer et al, 2014[8]
**DB13-041**	c.222_223dupGC	p.Pro75Argfs*118	Frameshift	Y	Y	Seltzer et al, 2014[8]
**DB13-052a1**	c.460dupG	p.Glu154Glyfs*301	Frameshift	Y	Y	Seltzer et al, 2014[8]
**DB13-052a2**	c.460dupG	p.Glu154Glyfs*301	Frameshift	Y	Y	Seltzer et al, 2014[8]
**DB14-031**	c.263_278del16		Frameshift	Y	N	-

Table e-1. Genotype of patients with *FOXG1* mutations.

Patient ID	OFC	Independent ambulation	Spoken language	Epilepsy	MRI findings	CSF Neurotransmitters
**DBL01-01**	< -3 SD	N	N	Y (GTCS, Focal)	ADCC, FTU, DM	Normal
**DBL01-02**	< -3 SD	N	N	Y (IS, GTCS)	ADCC, FTU, CH, DM	Normal
**DBL01-03**	< -3 SD	N	N	Y (GTCS)	ADCC, FTU, DM	Normal
**DBL01-04**	-3 SD to -2SD	N	N	N	ADCC, FTU, DM	Normal
**DBL01-05**	-3 SD	N	N	N	ACC, DM	Normal
**DBL01-06**	< -3 SD	N	N	Y	ADCC, FTU, CH, DM	Normal
**DBL01-07**	< -3 SD	N	N	Y	ADCC, FTU, CH, DM	Normal
**DBL01-08**	-3 SD	N	N	Y	ADCC, FTU, DM	Low HVA, low biopterin
**DBL01-09**	- 1.33 SD	Y	Y	N (FC)	ADCC, FU, CH, DM	Low HVA and 5-HIAA
**DBL01-010a1**	0 SD	Y	Y	Y (Absence, GTCS)	Posteriorly deficient CC (parietal underdevelopment), CH, NM	Normal
**DBL01-010a2**	+ 0.67 SD- +1.33 SD	Y	Y	N (FC)	Normal CC , Normal cerebellum, NM	Normal
**DBL01-010a3**	+1.33 SD- +2 SD	Y	Y	Y (tonic, absences, focal)	Posteriorly deficient CC (parietal underdevelopment), mild CH, NM	Normal
**DB12-001**	No info	N	N	Y (Tonic, myoclonic)	No MRI	Not done
**DB12-002**	-5 SD	N	N	Y (GTCS)	CC hypoplasia; FU; CH, MNA	Not done
**DB12-003**	+MIC	N	N	N	ACC, FU, MNA	Not done
**DB12-004**	-4 SD	N	N	Y (Complex partial)	ADCC, MNA	Not done
**DB12-006**	-4 SD	N	N	Y (tonic, GTCS)	No MRI	Not done
**DB12-008**	+MIC	N	N	Y (tonic)	CC hypoplasia, FU, MNA	Not done
**DB12-016**	+MIC	N	N	Y (Tonic)	ADCC, MNA	Not done
**DB12-017a1**	-4 SD	N	N	Y (Complex Partial)	CH, FU, MNA	Not done
**DB12-017a2**	-4 SD	N	N	N	CH, FU, MNA	Not done
**DB13-007**	-5 SD	N	N	Y (Myoclonic)	CC hypoplasia, FU, MNA	Not done
**DB13-029a1**	+MIC	N	N	Y (GTCS, Status Epilepticus)	No MRI	Not done
**DB13-029a2**	+MIC	N	N	Y (GTCS)	No MRI	Not done
**DB13-041**	-4 SD	N	N	Y (Tonic)	No MRI	Not done
**DB13-052a1**	-4 SD	N	Y	Y (Complex Partial)	No MRI	Not done
**DB13-052a2**	-4 SD	N	Y	Y (Complex Partial)	No MRI	Not done
**DB14-031**	-5 SD	N	N	Y (Tonic)	ADCC, FU, MNA	Not done

Table e-2. Clinical and neuroradiological findings in patients with *FOXG1* mutations. Abbreviations: 5-HIAA: 5-hydroxyindolacetic acid, ACC: agenesis corpus callosum, ADCC: anteriorly deficient corpus callosum, CC: corpus callosum, CH: cerebellar hypoplasia, CSF: cerebrospinal fluid, DM: delayed myelination, FC: febrile convulsions, FU: frontal underdevelopment, FTU: frontotemporal underdevelopment), GTCS: generalised tonic clonic seizures, IS: infantile spasms, +MIC: microcephaly present but actual head circumference not available, MNA: myelination not assessed, MRI: magnetic resonance imaging, N= No, NM: normal myelin, OFC: occipitofrontal circumference, SD= standard deviation, Y= Yes

Patient ID	Involuntary Movements	Age of Onset	Body Region Involved	Functional Interference	Clinical course	Hospitalisation due to movements
**DB12-001**	Yes	Birth	Generalised	Toileting, dressing, sleep, eating, other (bath)	3	No
**DB12-002**	Yes	Birth	Generalised, face	Dressing, sleep, eating, other (play, learning, communication with device)	3	No
**DB12-003**	Yes	<12 months	Mouth/tongue, arms/hands	Dressing, eating, other (non-verbal communication)	3	No
**DB12-004**	Yes	4 months	Generalised, face	Toileting, dressing, sleep, eating, speaking, other (walking)	4	No
**DB12-006**	Yes	<12 months	Generalised, trunk	Sleep, eating, other (sitting)	5	No
**DB12-008**	Yes	10 months	Generalised, trunk	Toileting, dressing, eating, other (play, communication with device)	3	No
**DB12-016**	Yes	12 months	Arms/hand, legs/feet	eating	4	No
**DB12-017a1**	Yes	3-5 months	Generalised	Toileting, dressing, sleep, eating, speaking, other (fine motor tasks, walking)	2	No
**DB12-017a2**	Yes	12 months	Generalised, trunk	Dressing, sleep, eating, speaking, other (fine motor tasks)	4	No
**DB13-007**	Yes	9 months	Generalised, face, trunk	Toileting, dressing, sleep, eating	4	No
**DB13-029a1**	Yes	6 months	Arms/hands, legs/ feet	Toileting, dressing, sleep, eating	3	No
**DB13-029a2**	Yes	3 months	Arms/hands, legs/feet	Dressing, sleep	3	No
**DB13-041**	Yes	Birth	Generalised	Toileting, dressing, eating, other (sitting, walking, standing)	3	No
**DB13-052a1**	Yes	12 months	Generalised, trunk	Dressing, eating, speaking, other (pushing up, walking)	5	No
**DB13-052a2**	Yes	12 months	Generalised, trunk	Dressing, eating, speaking, other (pushing up, walking)	5	No
**DB14-031**	Yes	Unknown	Generalised, trunk	Dressing, eating, speaking, other (pushing up, walking)	3	No

Table e-3. Parental perception on the impact of movement disorders on everyday activities, response to therapeutic interventions and evolution over time. Key: 1= much improved, 2= improved, 3= stable, 4= worsened, 5= much worse**.** When describing involuntary movements, the term ‘generalised’ indicates involvement of the mouth/tongue, head/neck, arms/hands and legs/feet**.**
