# Supplementary material for: Delineation of the movement disorders associated with FOXG1 mutations
Source: Neurology. 2016 May 10;86(19):1794–800. doi: 10.1212/WNL.0000000000002585 (PMC4862244; doi:10.1212/WNL.0000000000002585)
Supplement: Data Supplement [file supp_WNL.0000000000002585_Figure_e-1.pdf]

Figure e-1. Brain magnetic resonance imaging findings.

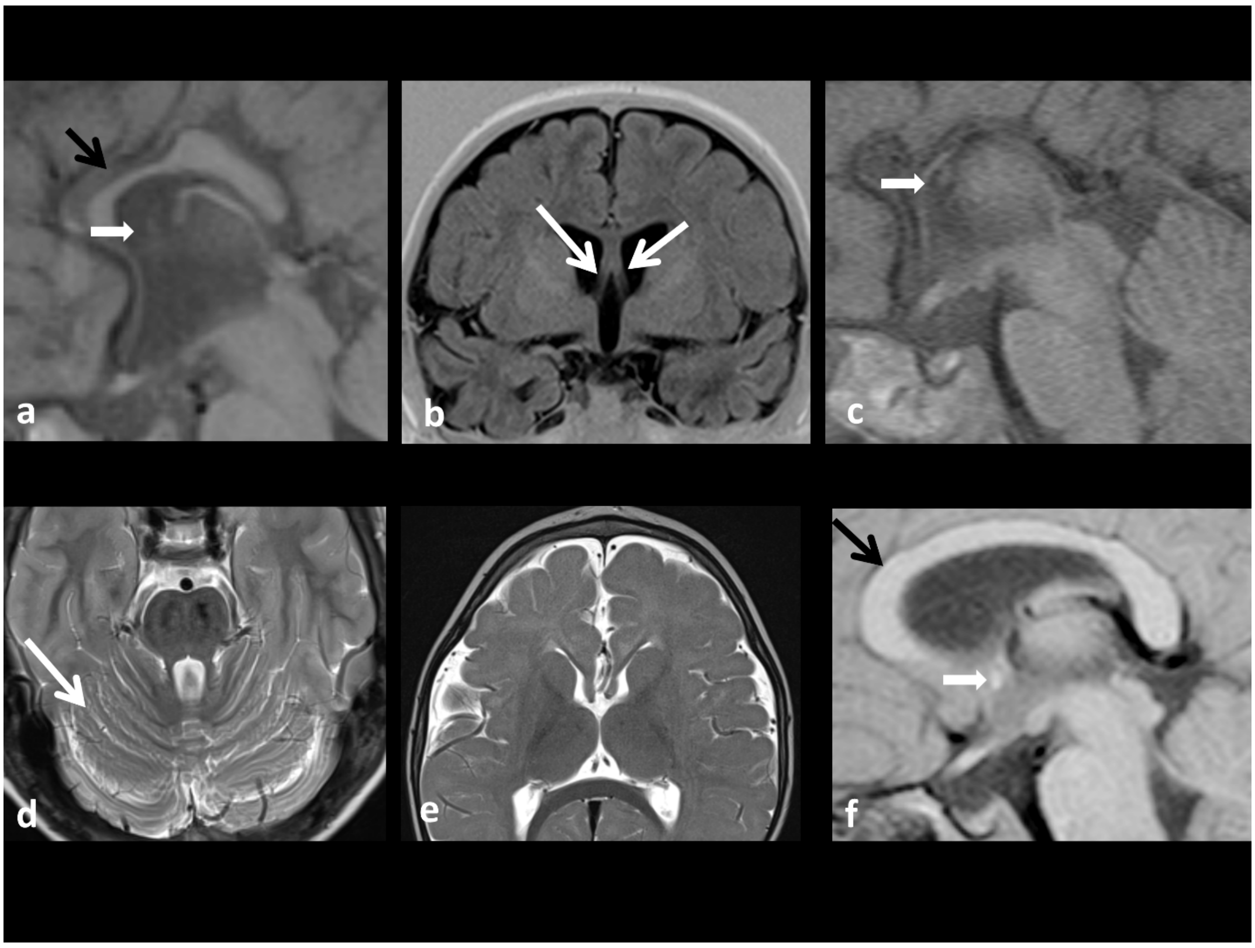

a) Sagittal T1 –weighted image on patient DBL01-02 demonstrating under-development of the frontal lobe with an anteriorly deficient corpus callosum (black arrow) and hypoplasia of the anterior commissure (white arrow); b) Coronal IR-weighted image on case DBL01-03 showing thickened fornices (white arrows); c) Sagittal T1-weighted image on patient DBL01-05 showing agenesis of the corpus callosum and a hypoplastic anterior commissure (white arrow); d) Axial T2 weighted sequence in case DBL01-09 showing cerebellar atrophy with widened cerebellar fissures (white arrow); e) Axial T2-weighted image in case DBL01-05 at 13 months showing generally delayed maturation of myelination; Basal ganglia have normal appearance. f) Sagittal T1-weighted weighted image in an unaffected 23 month old child showing the normal configuration of the corpus callosum (black arrow) and the normal anterior commissure (white arrow).
