# Supplementary material for: Delineation of the movement disorders associated with FOXG1 mutations
Source: Neurology. 2016 May 10;86(19):1794–800. doi: 10.1212/WNL.0000000000002585 (PMC4862244; doi:10.1212/WNL.0000000000002585)
Supplement: Videos [file supp_86_19_1794_v2_index.html]

Videos 

# Delineation of the movement disorders associated with *FOXG1* mutations

## Data Supplement

Eight videos and legends; eight .mp4 files and one PDF file.

**Neurology® data supplements are not copyedited before publication. Published editorials and translations have been copyedited.  
 © 2016 American Academy of Neurology.  
  
 Files in this Data Supplement:**

- Video legends - PDF file
- Video e-1 - .mp4 file
- Video e-2 - .mp4 file
- Video e-3 - .mp4 file
- Video e-4 - .mp4 file
- Video e-5 - .mp4 file
- Video e-6 - .mp4 file
- Video e-7 - .mp4 file
- Video e-8 - .mp4 file
