# Supplementary material for: Delineation of the movement disorders associated with FOXG1 mutations
Source: Neurology. 2016 May 10;86(19):1794–800. doi: 10.1212/WNL.0000000000002585 (PMC4862244; doi:10.1212/WNL.0000000000002585)
Supplement: Videos [file supp_WNL.0000000000002585_Video_legends.pdf]

Video Legends

Video 1. Chorea/ athetosis in *FOXG1* mutations. Three patients are shown. DBL01-010a2: choreoathetosis of the upper limbs, dystonia of the upper limbs and myoclonus. DBL01-010a1: choreoathetosis of the lower limbs (involving the feet and toes), which mildly impairs tandem gait. DB13-052a1: prominent distal lower limb choreoathetosis, generalized dystonia, limited functional hand use and stereotypies (body rocking).

Video 2. Dystonia in *FOXG1* mutations. Five patients are shown. DBL01-07: generalized dystonia, including anterocollis and striatal toe. DB13-029a1: generalized dystonia with retrocollis. DB13-041: generalized dystonia with brief jaw opening dystonia. DB12-006: distal lower limb dystonia. DBL01-09: distal arm/ hand posturing and toe strike during ambulation.

Video 3. Orofacial dyskinesias in *FOXG1* mutations. 6 patients are shown. DBL01-01: chewing movements and tongue protrusion. DBL01-07: facial grimacing and lip pursing. Patients DB12-004, DB13-041, DB13-052a1 and DB13-052a2 exhibit repetitive mouth opening movements resembling oromandibular dystonia.

Video 4. Facial Dystonia in *FOXG1* patients. One patient is shown, DB13-029a2: facial dystonia with reduced facial expression and eye blinking. Forehead wrinkling, axial and upper limb dystonia are also shown.

Video 5. Stereotypies in *FOXG1* mutations. 6 patients are shown. DBL01-01 and DBL01-06: hand grasping movements, which are mainly unilateral and do not involve bringing the hands to the midline. DB13-029a1: midline, hand stereotypies (wringing), limb dystonia, and retrocollis. DB12-017a2 and DB13-007: lower limb (pedalling) stereotypies. DB13-052a1: constant body rocking.

Video 6. Myoclonus and dystonia in *FOXG1* mutations. One patient is shown. DBL01-010a3: generalized choreoathetosis, distal upper limb dystonic posturing and myoclonic jerks of the upper limbs and trunk.

Video 7. Response to levodopa in *FOXG1* mutations. One patient is shown. DBL01-09: At the age of 14 years, prior to levodopa administration, there is distal upper limb dystonic posturing, chorea, distal tremor and subtle myoclonic jerks affecting the trunk and limbs. At the age of 17 years, whilst on levodopa, these movements have much improved but some distal upper limb posturing (left more than right) and tremor still remains.

Video 8. Variable movement disorder severity in siblings with *FOXG1* mutations. Three siblings, DBL01-010a1, DBL01-010a2 and DBL01-010a3, are shown. There is different movement disorder severity, with DBL01-010a3 more severely affected (exhibiting limb dystonia, choreoathetosis and myoclonic jerks) than the other siblings.
